# Cellular and molecular characteristics of the premetastatic niches

**DOI:** 10.1002/ame2.12356

**Published:** 2023-10-30

**Authors:** Hongfei Liu, Guoxin Zhang, Ran Gao

**Affiliations:** ^1^ Department of Otolaryngology, Head and Neck Surgery Beijing Tongren Hospital, Capital Medical University Beijing China; ^2^ National Human Diseases Animal Model Resource Center, The Institute of Laboratory Animal Science Chinese Academy of Medical Sciences & Peking Union Medical College Beijing China; ^3^ NHC Key Laboratory of Human Disease Comparative Medicine Beijing Engineering Research Center for Experimental Animal Models of Human Critical Diseases Beijing China; ^4^ Beijing Engineering Research Center for Experimental Animal Models of Human Critical Diseases Beijing China

**Keywords:** extracellular vesicles, immunosuppression, premetastatic niches, tumor metastasis

## Abstract

The premetastatic niches (PMN) formed by primary tumor‐derived molecules regulate distant organs and tissues to further favor tumor colonization. Targeted PMN therapy may prevent tumor metastasis in the early stages, which is becoming increasingly important. At present, there is a lack of in‐depth understanding of the cellular and molecular characteristics of the PMN. Here, we summarize current research advances on the cellular and molecular characteristics of the PMN. We emphasize that PMN intervention is a potential therapeutic strategy for early prevention of tumor metastasis, which provides a promising basis for future research and clinical application.

## Graphical abstract

The main components and mechanisms of the premetastatic niches (PMN). Extracellular vesicles secreted by tumor enter the circulation and bone marrow, mobilizing immune cells to reach distant organs, regulate the accumulation of immune cells, and form an inhibitory immune niche. PMN mainly includes immune cells, cytokines, chemokines, and ECM.

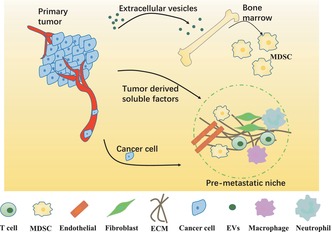

## 1 INTRODUCTION

Metastatic progression is the main factor of death in tumor patients, although the potential factors conducive to tumor metastasis remain unclear. Once established, metastasis is devastating. Before the colonization of the second organ, only a few proportion of the tumor cells that leave the primary tumor succeed in metastasis. Cancer cells are usually subjected to complex cascade processes, including shedding and intravasation of tumor cells, survival in the circulation, extravasation, settlement, and growth in the secondary organ. Clarifying potential mechanisms may help us identify strategies and therapeutic targets for the prevention of cancer metastasis that would benefit patients.

The “seed‐and‐soil” hypothesis contributes to our understanding of tumor metastasis. Metastatic tumor cells (“seeds”) arrive at specific organ sites (“soil”) where the niche is conducive to metastasis. An increasing number of studies suggest that primary tumors can facilitate colonization by inducing the formation of a supportive niche (called premetastatic niches [PMN]) at secondary organ sites. In recent years, the role and significance of PMN have gained increasing attention.^1^

It has been found that to make distant organs suitable for the colonization of tumor cells, PMN establishes complex mechanisms within the metastasis site. The progress of these studies provides good knowledge for clarifying the mechanism of early tumor metastasis and designing more favorable diagnostic and therapeutic methods. Accordingly, this article aims to summarize the cellular and molecular characteristics of PMN formation, which helps explain the mechanism of early metastasis.

## 2 MOLECULAR COMPONENTS

### 2.1 Soluble factor and tissue factor

The identification of tumor‐derived secretory factors will be discussed. These secretory factors demonstrate the ability to produce the PMN in different organs, as well as the cellular and molecular changes in different organs through their actions.

#### 2.1.1 Chemokine

Chemokines play an irreplaceable function in regulating the PMN. Tumor‐associated macrophages (TAMs) and regulatory T cells (Treg) are recruited by chemokine C–C motif ligand 2 (CCL2) from primary tumor cells to form PMNs in the lung.^2^ Th2 cytokines induce complement 3 in lung mesenchymal stromal cells, and their expression promotes the recruitment of neutrophils to stimulate PMN formation.^3^ Vascular endothelial growth factor (VEGF) secreted by colorectal cancer cells stimulates TAMs to generate chemokine (C–X–C motif) ligand 1 (CXCL1), which accumulates myeloid‐derived suppressor cells (MDSCs)into the liver tissue. MDSCs promote PMN formation and liver metastasis.^4^ In conclusion, targeting chemokine signal transduction in the PMN is a potential therapeutic strategy to prevent tumor metastasis.

#### 2.1.2 Exosomes (protein and noncoding RNA) or extracellular vesicles

Exosomes are extracellular vesicles (EVs) with a size of 30–150 nm,^5^ which contain a large number of proteins and nucleic acids from cells.^6^ Exosomes from different cells and tissues have an “innate” secretion tendency of distributing to premetastatic sites, such as the lung, the liver, bone marrow, and the brain, which reflects the organotropism of releasing cells.^7^ Chemotherapy‐elicited EVs are enriched in annexin A6 (ANXA6), which promotes the activation of nuclear factor (NF)–κB‐dependent endothelial cells and the polarization of Ly6C^+^CCR2^+^ monocytes in the pulmonary PMN.^8^ EVs rich in miR‐181a‐5p secreted by colorectal cancer cells continuously activate hepatic stellate cells (HSCs) by targeting suppressors‐of‐cytokine‐signaling 3 and activating interleukin‐6 (IL‐6)/STAT3 signaling pathways. The chemokine C–C motif ligand 20 (CCL20) derived from activated HSCs further activates the CCL20/CCR6/ERK (extracellular regulated protein kinases)1/2/ELK (transcription factor involved in ERK‐induced cellular proliferation)‐1/miR‐181a‐5p positive feedback loop, leading to the formation of PMN.^9^ MiR‐21, secreted by the in situ implantation of SCP28 breast tumor cells, promotes osteoclast formation and osteopenia by regulating PDCD4 protein, thus accelerating bone damage to reconstruct the microenvironment for bone Metastasis, which contributes to the formation of the PMN.^10^ Collectively, targeting the exosome to prevent the formation of the PMN may become a therapeutic method to prevent progression of the tumor in the future.

#### 2.1.3 Extracellular matrix components

The extracellular matrix (ECM) forms a scaffold that supports the attachment of tumor cells and the reactivation of survival signals. The structure of the intrinsic ECM of an organ is usually not conducive to supporting the attachment and movement of tumor cells. Therefore, the reconstruction of ECM is an important step in PMN formation.^11^, ^12^ Among ECM components, fibrinogen deposition plays an indispensable role in the formation of the PMN. The EVs of melanoma cells with a low expression of IGF2 mRNA binding protein 1 inhibit the deposition of fibronectin and the aggregation of CD45^+^ cells in the lung, thus blocking PMN formation.^13^ Matrix metalloproteinases (MMPs) can target the ECM protein, which is one of the characteristics of PMN formation.^14^ Lysine oxidase‐like protein 2 (LOXL2) from liver cancer enhanced the recruitment of CD11b^+^/CD45^+^ BMDCs (bone marrow‐derived cells) in the lung and significantly increased MMP9 and fibronectin in lung fibroblasts. LOXL2‐induced matrix stiffening synergistically regulated the formation of pulmonary PMN.^15^ Similarly, under hypoxic conditions, exosomes secreted by prostate cancer cells increased the levels of MMP2, MMP9, fibronectin, and collagen and upregulated the number of CD11b^+^ cells at the PMN, promoting distant metastasis.^14^ Periosteal protein (POSTN) is one of the components of the ECM, which interacts with fibronectin, tendinosin‐c, and collagen I, IV, and V to exert function.^16^ POSTN promotes the accumulation of pulmonary MDSCs in early breast cancer. POSTN detected in MDSCs showed that the activation of ERK, AKT, and STAT3 decreased the number of neutrophils and monocytes in the bone marrow of mice and inhibited the recruitment of MDSCs to the PMN.^17^ However, more systematic research is needed to reveal these complex and confusing mechanisms.

## 3 THE FORMATION AND CHARACTERISTICS OF PMN

Tumor cells need favorable environments with nutrients, immune cells, and the ECM to successfully colonize distant organs. They are separated from the primary tumor and enter the circulation, but due to factors such as immune system attacks, only a few of the tumor cells finally enter the distant host organs to form metastasis.^18^, ^19^ Therefore, metastatic organs need to construct an immunosuppressive niche to favor the survival of circulating tumor cells (CTCs) and prevent the attack of natural killer (NK) cells, CD4^+^ and CD8^+^ T cells. Protumor immune cells are important components of the PMN, such as MDSCs, Treg, TAMs, and tumor‐associated neutrophils, which are recruited into host organs to form an immunosuppressive niche.^20^, ^21^ The PMN is initiated by the primary tumor through the secretion of factors, which facilitates the successful colonization of metastatic tumor cells. The PMN has the following five characteristics. These characteristics are related to whether tumor cells can survive or remain dormant after reaching the metastatic site.

### 3.1 Vascular alteration

Vascular alteration is a salient characteristic of PMN formation. It has been shown that many factors promote angiogenesis and permeability of tumor microenvironment (TME) to increase metastasis.^22^ Epidermal proteins in EVs can promote angiogenesis by upregulating the expression of VEGF and VEGFR1 in pulmonary vascular endothelial cells.^23^ Similarly, Zeng et al.^24^ demonstrated that tumor‐derived exosomes miR‐25‐3p favor the formation of the PMN and support colorectal cancer metastasis by inducing angiogenesis and permeability.^24^ Peinado et al.^25^ further demonstrated that the EVs derived from B16–F10 (mouse skin melanoma cell line) increase pulmonary vascular permeability and initiate PMN formation, which improves the capability of spontaneous lung and bone metastasis. Using proteomics, the EVs from breast cancer were identified as rich in nucleoside diphosphate kinase (NDPK), and the mice treated with EVs showed pulmonary vascular leakage, indicating that NDPK regulates the host microenvironment to facilitate the formation of the PMN and NDPK inhibitors attenuate metastasis.^26^ The vasculature is crucial for multistage metastasis, including the entry of cancer cells to the metastatic organs and providing nutritional support for cancer cells.^27^ Importantly, more research is needed to understand the function of the vasculature in PMN formation.

### 3.2 Immunosuppression

The surveillance of immune system not only plays a key role in the tumor progression but also the immune status has a significant impact on PMN formation. Tumor cells survive through the established immunosuppressive niche to protect themselves from apoptosis. Immunosuppression in the PMN involves the regulation of immune cells by regulatory factors contained in tumor‐secreted EVs. EVs secreted by pancreatic cancer cells contain immunosuppressive factors that inhibit NK‐cell function, which is conducive to the formation of the PMN.^28^ IL‐6/STAT3 signaling is aberrantly activated for orchestrating PMN formation and immunosuppression in lung metastasis.^29^ EVs secreted by melanoma shuttled tumor antigens to lymphatic endothelial cells for cross‐presentation on major histocompatibility complex‐I, leading to the induction of antigen‐specific CD8^+^ T‐cell apoptosis.^30^ EVs derived from Lewis lung cancer cells promote the secretion of chemokine (C–C motif) ligand 1 (CCL1) by pulmonary fibroblasts, activating specific receptor (C–C motif) receptor 8 (CCR8), and induce Treg differentiation, which ultimately contributes to the establishment of immunosuppressive PMN.^31^ Together, the EVs released by primary tumor cells are crucial to the immune regulation of the PMN, and the immunosuppression of the PMN ultimately promotes tumor metastasis.

### 3.3 Inflammation

Increasing evidence supports the involvement of inflammation in different steps of cancer development, including cancer initiation and progression.^32^ Rodrigues et al.^33^ found that the exosomes secreted by tumors contained CEMIP (cell migration‐inducing and hyaluronan‐binding protein) protein, which regulated brain endothelial cells and microglia. CEMIP induces endothelial cells and inflammation in perivascular niches and promotes tumor brain metastasis by upregulating pro‐inflammatory cytokine tumor necrosis factor (TNF), prostaglandin–endoperoxide synthase 2 (PTGS2), and CCL/CXCL.^33^ Sphingosine‐1‐phosphate (S1P) generated by tumor‐induced sphingosine kinase 1 (SphK1) in the lung PMN increases the recruitment of macrophages into the lung and induces IL‐6 and important signal pathways for lung metastasis and colonization. Obesity‐related inflammation increases the expression of S1P, acts on S1PR1, promotes macrophage infiltration and tumor progression, and confirms the role of circulating S1P produced by tumor and SphK1/S1P/S1PR1 axis in inflammation and tumor metastasis.^34^ Chronic inflammation is an important driving factor for tumor progression. Improper activation of toll‐like receptor 4 (TLR4) and other sensory receptors in immune cells can lead to unresolved inflammation, contributing to tumor progression.^35^ Leukotriene secreted by neutrophils can also contribute to tumor metastasis by accumulating in the PMN.^36^ Neutrophil inflammation driven by TLR4 and myeloid differentiation factor 88 increases the metastasis of melanoma cells to the lung and lymph nodes.^37^ In conclusion, further exploration is needed to investigate the role of inflammation‐related factors in the formation of the tumor PMN.

### 3.4 Organotropism

The organotropism characteristics of tumor metastasis are related to the PMN, because some types of cancer are easy to metastasize to specific organs with selective niches. Cancers disseminate to lymph nodes, the lung, bone, and the liver with different frequencies. Tumor cells release exosomes to establish a niche conducive to metastasis in organs. Tumor‐derived exosomes remain in the organs with cancer cell metastasis, promoting the formation of the PMN.^38^

Lung metastasis is one of the most common metastatic sites in patients, and most of our research on PMN biology is based on the study of lung metastasis.^39^ In lung PMN, the exosomes enrich the accumulation of integrin in the lung microenvironment to enhance the expression of S100A4 in fibroblasts to establish pro‐inflammatory PMN.^38^ Nicotinamide phosphoribosyltransferase secreted by the primary tumor activates neutrophils accumulated in the lung to form neutrophil extracellular traps (NETs) through SIRT1, which facilitates PMN formation and lung metastasis of breast cancer.^40^ In brain metastasis of lung cancer, nicotine exposure recruits STAT3‐activated N2 neutrophils in the brain PMN, leading to the secretion of miR‐4466, which promotes brain tumor stem cells through the SKI/SOX2/CPT1A axis, thereby promoting metastasis.^41^

Bone is a common metastatic site in many solid tumors.^42^ Primary tumors and their derived circulating factors promote PMN formation and metastasis cell colonization by regulating the target cells resident in bone.^43^ RANK/RANKL signal can induce osteolytic and immunosuppressive microenvironment in bone by inducing osteoclast generation and Treg amplification, thus prompting the formation of the PMN.^44^, ^45^ Subsequently, several studies further proved that miR‐19a and miR‐20a‐5p favor osteoclast proliferation and differentiation, cause bone damage and tissue remodeling, and regulate the generation of the PMN.^46^, ^47^ However, the more detailed mechanism needs further research and discussion.

The formation of the liver PMN occurs at the initial stage of visceral metastasis. In 2015, Costa‐Silva et al. conducted animal experiments to show that Kupffer cells receiving exosomes from pancreatic ductal adenocarcinoma (PDAC) caused TGF‐β secretion and fibronectin expression of HSCs were upregulated, thus inducing the formation of niches before liver metastasis. Among them, macrophage migration inhibitory factor in exosomes plays a major role, which may be a prognostic marker for the development of PDAC liver metastasis.^48^ Recently, the same study has found that in KPC (mouse model of pancreatic ductal adenocarcinoma) mice, the EVs of PDAC promote the enrichment of macrophages in the liver niches. It has been proved that the overexpression of Rab27a is related to the poor prognosis of cancer and may be related to the extensive remodeling caused by the release of systemic EVs, which contributes to the biogenesis of EVs.^49^ The Rab27a GTPase is overexpressed in advanced cancer. However, these EVs could not prevent the decrease of myeloid cell infiltration in the liver of mice transplanted with Rab27a‐deficient KPC cells, which indicated that Rab27a had other mechanisms in PMN formation besides the one mediated by EVs.^50^ In short, the PMN in different metastatic target organs exhibits complex molecular and cellular components, and its detailed molecular mechanism needs to be further clarified.

### 3.5 Metabolic reprogramming

Metabolic reprogramming has been shown to be involved in the formation of the PMN, including glucose metabolism, lipid metabolism, and oxalate metabolism. Fong et al.^51^ found that tumor cells can inhibit pyruvate kinase and glucose uptake by secreting vesicles carrying high levels of miR‐122 in the PMN. Glucose metabolism is also associated with macrophage‐acquired immunosuppressive phenotype. Morrissey et al.^52^ demonstrated that tumor‐derived exosomes increase PD‐L1 expression through NF‐κB‐dependent glycolytic reprogramming and promote macrophages to polarize toward immunosuppressive phenotype.

Lipid metabolism can also promote the formation of PMN. Zhang et al.^53^ proved that HSPC111 is a major upregulated gene in HSCs incubated with exosomes derived from CRC (colorectal cancer) cells. HSPC111 changes the lipid metabolism of fibroblasts by phosphorylating ATP citrate lyase, upregulates the level of acetyl CoA, increases the acetylation of H3K27 in cancer‐associated fibroblasts (CAFs), promotes the expression and secretion of C–X–C motif chemokine 5 (CXCL5), and affects the formation of the PMN and liver metastasis of colorectal cancer.^53^

Zeng et al.^54^ proved that oxalate can induce the formation of NET by activating NADPH oxidase in the lung and promoting the PMN formation of breast cancer. Pharmacological inhibition of hydroxyacid oxidase 1 can effectively inhibit the accumulation of oxalic acid in the lung caused by a primary tumor.^54^ These results indicate that systemic metabolism can be reprogrammed to promote disease progression by changing the utilization of glucose, lipid, and oxidase involved in PMN.

## 4 COMPONENTS INVOLVED IN THE PMN

**FIGURE 1:**
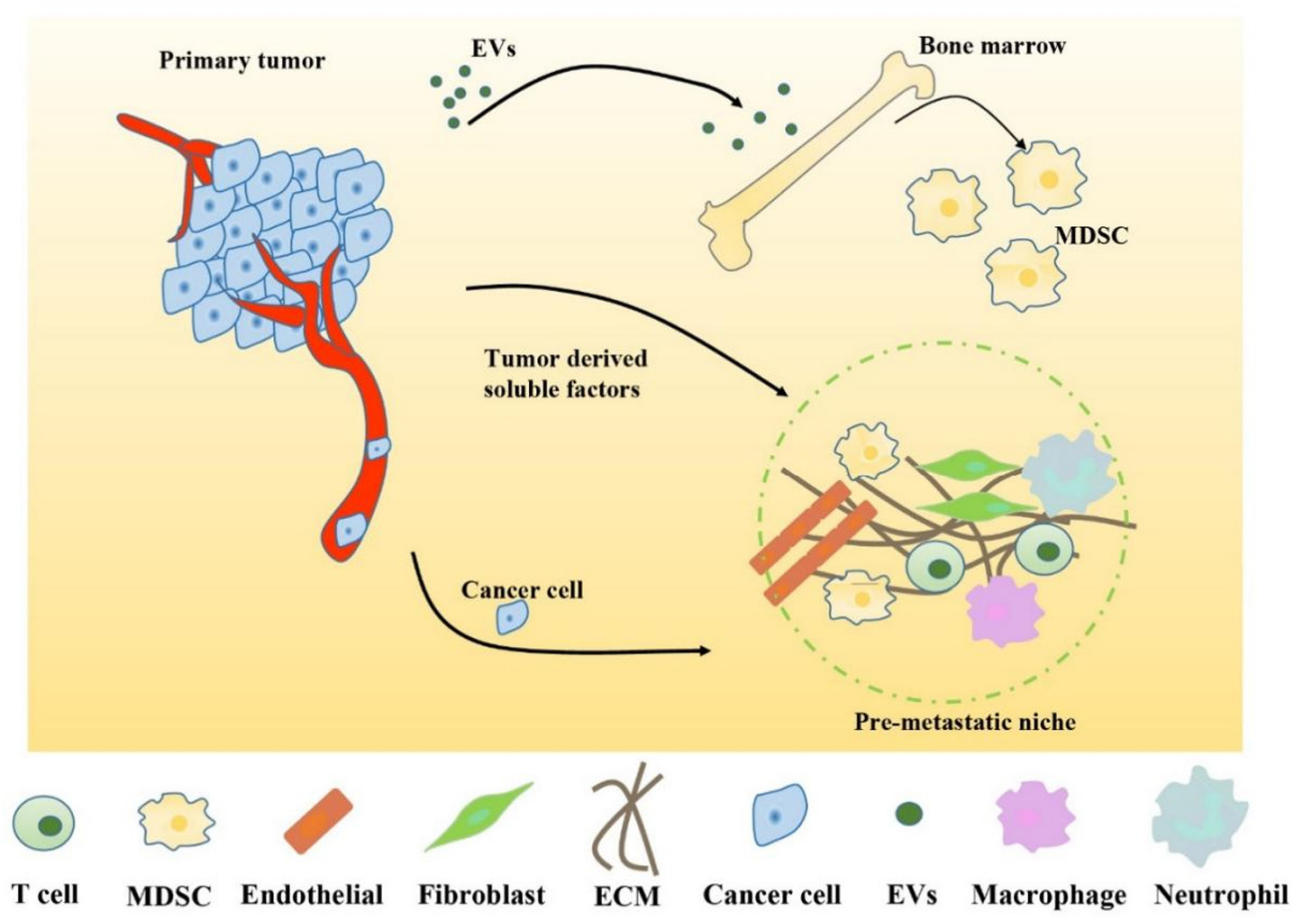
The main components and mechanisms of the PMN. EVs secreted by the tumor enter the circulation and bone marrow, mobilizing immune cells to reach distant organs, regulate the accumulation of immune cells, and form an inhibitory immune niche. The PMN mainly includes immune cells, cytokines, chemokines, and the ECM. ECM, extracellular matrix; EVs, extracellular vesicles; MDSC, myeloid‐derived suppressor cell; PMN, premetastatic niche.

The PMN is established through complex cellular and molecular changes, which ultimately create a favorable microenvironment, and the transferred cells can be colonized and survive at an appropriate time.^55^ Figure 1 summarizes the components and mechanisms related to PMN formation.

### 4.1 Cellular components

#### 4.1.1 Myeloid‐derived suppressor cells

Tumor‐derived components are considered crucial factors in the initiation of the PMN, which involves crosstalks between tumor‐derived factors and tumor‐mobilized BMDCs.^20^ MDSCs are a group of heterogeneous immature immune cells derived from BMDCs, which play a key role in the development of cancer by suppressing immune effector cell functions and contributing to the formation of PMN.^20^, ^56^, ^57^ Antagonists of the S100A8‐mediated TLR4/MD‐2 complex can inhibit the recruitment of MDSCs in premetastatic lung.^58^ Bone marrow–derived Gr‐1^+^ myeloid cells express thrombospondin‐1 to inhibit metastasis.^59^ In another study, Gr1^+^ CD11b^+^ immature myeloid cells decrease interferon‐γ (IFN‐γ) and elevate Th2 cytokine production in premetastatic lungs.^60^ Granulocyte MDSCs increase vascular permeability by secreting MMP‐9 and IL‐10 in the PMN.^61^ Primary tumor hypoxia provides cytokines and growth factors to build a PMN through the accumulation of MDSCs and inhibit the cytotoxic effect function of the NK‐cell population.^62^ In conclusion, multiple factors can promote the transfer of MDSCs to organs by mobilizing them to specific second organs and establishing a favorable microenvironment for transfer.

#### 4.1.2 Fibroblasts

Fibroblasts found in primary and metastatic tumors are called cancer associated fibroblasts (CAFs), which play a key role in PMN formation. The tumor cell educates fibroblasts to construct tumor‐supported host matrix through STAT3 signal and c‐Jun N‐terminal kinase signal pathway, promotes ECM degradation and reconstruction, and stimulates PMN formation.^63^, ^64^ LncSNHG5‐ZNF281‐CCL2/CCL5 signal axis promotes angiogenesis and vascular permeability in CAFs and plays a key role in PMN formation in breast cancer.^65^ The colonization of specific metastatic organs enables metastasis‐related fibroblasts to have an inflammatory phenotype. For example, EVs derived from cancer cells activate fibroblasts to form the PMN by secreting TNF, IL‐6, IL‐8, IL‐1α, and IL‐1β.^64^, ^66^, ^67^, ^68^ Research on the formation of the CAF in the PMN and its tumor‐promoting function has found many mechanisms, thus proposing a variety of potential therapeutic targets. These findings target CAF or related signaling pathways for drug development.

#### 4.1.3 Macrophages

The M1/M2 macrophage paradigm plays different functions in tumor progression. It mainly codisplays M1‐like antitumor phenotype and M2‐like tumor‐promoting phenotype commonly considered as TAMs, and their importance in metastasis has been confirmed.^69^ Colorectal cancer‐derived exosome miRNA‐934 promotes macrophage polarization into M2 macrophages and the formation of the PMN in colorectal cancer.^70^ Exo‐miR‐519a‐3p activates the mitogen‐activated protein kinase/ERK pathway by targeting dual specificity phosphatase 2, which leads to M1 polarization to M2‐like macrophages and accelerates liver metastasis of gastric cancer by inducing angiogenesis and promoting the formation of the PMN in the liver.^71^

#### 4.1.4 Neutrophils

Neutrophils account for about 80% of all white blood cells, which is essential to control infection. Circulating neutrophilism is associated with poor prognosis in patients with different cancers.^72^ Numerous studies found that neutrophils are irreplaceable contributors to the formation of the PMN.^54^, ^73^ In IFN knockout mice, a higher metastatic load was shown, accompanied by a large number of neutrophils accumulating in the lungs. MMP9, S100A8/A9 and increasing expression of neutrophils in lung infiltration, help promote the formation of PMN and more effectively support tumor cell survival and proliferation in metastatic organs.^74^ Ovarian tumor–derived inflammatory factors stimulate neutrophils to extrude chromatin reticulum called neutrophils extracellular traps (NET), and NET deposition promotes tumor cell proliferation and immunosuppression.^75^, ^76^ In addition, the accumulation of neutrophils within the PMN provides an immunosuppressive niche characteristic of decreased infiltrating CD8^+^ T cells and upregulated PD‐L1.^77^, ^78^, ^79^ However, the signal of its influence on the mechanism of PMN immunosuppression needs further study.

#### 4.1.5 Endothelial cells

Intravasation and extravasation are necessary to the progress of the metastatic cascade.^80^ Therefore, an endothelial barrier is very important in preventing tumor dissemination. It is proposed that altered endothelial cells directly influence inflammation and tumor metastasis.^81^ Endothelial cells induce tumor cell growth by forming blood vessels, guide tumor cells to spread in blood or lymph, and play a major role in promoting the formation of metastasis. Tumor‐resident bacteria *Escherichia coli* disrupt the gut endothelial barrier; upon endothelial barrier impairment, bacteria disseminate to the liver and improve the formation of a PMN.^82^ EMCN (Endomucin) is a transmembrane *O*‐sialylated protein expressed on the surface of endothelial cells. Zhang et al.^83^ proved that the lack of endothelial EMCN will not significantly affect the growth of the primary tumor but strongly promote spontaneous metastasis. The mechanism of EMCN deficiency is mainly through the recruitment of Ly6G^+^ neutrophils and upregulation of MMP9, S100A8/A9, and TGF‐β expression, leading to the formation of the PMN. Further investigation is needed to determine whether endothelial cells also play a crucial role in a different PMN organ.

#### 4.1.6 Cancer stem cells

A small number of pluripotent and mostly quiescent tumor cells, called cancer stem cells (CSCs), have been proved to be critical to the occurrence and development of tumors. Lin28B promotes primary tumors to produce more ALDH^+^ CSCs and secrete exosomes enriched in let‐7s, which can promote Lin28B recruitment of granulocytes and the transformation of N2 neutrophils. Then N2 neutrophils can form an immunosuppressive niche before lung metastasis through PD‐L2 upregulation and promote lung metastasis of breast cancer.^78^ Tumor stem cells in NSCLC (non‐small cell lung cancer) are stimulating factors of bone metastasis. Research shows that CSCs in NSCLC can promote bone metastasis by promoting adenosine production and osteoclast activity, producing an immunosuppressive PMN.^84^ TNF‐α in breast cancer stem cells induces vascular cell adhesion molecule 1 (VCAM‐1) to promote angiogenesis, and the formation of PMN which is induced by breast‐liver organ crosstalk.^85^ In conclusion, the role of CSC in PMN formation needs to be further clarified (Table 1).

**TABLE 1:** Summary of molecular and cellular components in PMN formation.

Donor cell	Molecular components	Tumor or stroma derived	Main functions and mechanisms	References
MDSCs	S100A8	Stroma	Changes the immune microenvironment and inhibits the recruitment of MDSCs	^58^
	Thrombospondin‐1	Stroma	Inhibits the recruitment of Gr1 (+) myeloid cells	^59^
	IFN‐γ	Stroma	Decreases IFN‐γ and elevates Th2 cytokine production	^60^
	IL‐10/MMP9	Stroma	Increases vascular permeability and immunosuppression	^61^
Fibroblasts	miR‐122	Tumor	Inhibits the glucose uptake of fibroblasts in PMN and increases the nutrition of tumor cells	^51^
	HSPC111	Tumor	Promotes the secretion of CXCL5, and CXCL5–CXCR2 axis enhances HSPC111, thus promoting the formation of PMN	^53^
	IL‐1α/IL‐1β	Tumor	Induces fibroblasts to secrete CXCL9/10 and form fibroblast niches	^64^
	Nidogen 1	Tumor	Promotes PMN formation by enhancing angiogenesis and the permeability of lung endothelial cells	^66^
	ITGBL1	Tumor	CRC‐derived Stimulates the TNFAIP3‐mediated NF‐κB signaling to activate fibroblast niches	^67^
Macrophages	miRNA‐934	Tumor	Induces M1‐like macrophages to form M2‐like macrophages, promoting PMN formation	^70^
	miR‐519a‐3p	Tumor	Induces M2‐like polarization of macrophages and promotes intrahepatic PMN	^71^
Endothelial cells	EMCN	Stroma	EMCN deficiency mainly affects the host microenvironment and leads to the lung PMN	^83^
Neutrophils	Interferon	Stroma	Lung infiltrating neutrophils facilitate an improved PMN formation	^74^
	PAD4	Stroma	Inhibits NETs and PMN formation	^75^
	Lin28B	Tumor	Enables the recruitment of neutrophils and N2 conversion, building an immune‐suppressive PMN	^78^
CSCs	let‐7s	Tumor	Forms an immunosuppressive PMN	^78^
	Adenosine	Tumor	Promotes immunosuppressive PMN	^84^
	TNFα	Tumor	TNFα treatment in breast cancer stem cells induces PMN formation	^85^
Other components				
Exosomes	Annexin A6	Tumor	Promotes the activation of endothelial cells, CCL2 induction, and Ly6C^+^CCR2^+^ monocyte expansion in the pulmonary PMN	^8^
	miR‐181a‐5p	Tumor	CCL20 activates the CCL20/CCR6/ERK1/2/Elk‐1/miR‐181a‐5p positive feedback loop, leading to the formation of PMN	^9^
	miR‐21	Tumor	miR‐21 derived from SCP28 cells facilitates the formation of PMN	^10^
Chemokine	CCL2	Tumor	Recruits TAMs and Treg to form PMN	^2^
	Complement 3	Stroma	Promotes the formation of NETs to promote PMN	^3^
	VEGF	Stroma	Stimulates TAMs to produce chemokine CXCL1, thereby recruiting MDSCs to form PMN	^4^

Abbreviations: CCL2, C–C motif ligand 2; CRC, colorectal cancer; CXCL1, chemokine (C–X–C motif) ligand 1; CXCR2, chemokine receptor 2; ELK, transcription factor involved in ERK‐induced cellular proliferation; EMCN, Endomucin; ERK, extracellular regulated protein kinases; IFN‐γ, interferon‐γ; IL, interleukin; MDSCs, myeloid‐derived suppressor cells; NET, neutrophil extracellular traps; NF, nuclear factor; PMN, premetastatic niche; TAMs, tumor‐associated macrophages; TNF, tumor necrosis factor; Treg, regulatory T cells; VEGF, vascular endothelial growth factor.

## 5 SIGNIFICANCE OF THE PMN FOR TUMOR METASTASIS INTERVENTION

### 5.1 Prevention of PMN formation

Blocking the formation of the PMN will be a promising strategy for taking early action on tumor metastasis. Methods to block the formation of the PMN include using loaded micellar nanoparticles,^86^ therapeutic treatment using miRNA‐enriched liposomes,^87^, ^88^ and reducing vascular permeability and ECM deposition.^89^, ^90^ Drugs loaded with micellar nanoparticles of tumor‐targeting peptides can inhibit metastasis. For example, hydrophilic low‐molecular‐weight heparin inhibits the recruitment of MDSCs by competitive binding with P‐selectin on the surface of endothelial cells, whereas hydrophobic all‐trans‐retinoic‐acid promotes exhaustion by inducing the differentiation of MDSCs. By regulating MDSCs, micellar nanoparticles can significantly ameliorate the inflammatory and immunosuppressive niche of lung and tumor sites and inhibit the formation of the PMN.^86^ Apart from exosomes, therapeutic miRNA‐enriched liposomes can be used for disease treatment by targeting pathological recipient cells.^87^ MiR‐29 shows effective antifibrosis activity by negatively regulating collagen expression. By designing a lung‐targeting liposomal nanovesicle delivery system to carry miR‐29a‐3p, this system significantly reduced the collagen I secretion of lung fibroblasts in vivo, thereby reducing the establishment of the PMN of tumor cells and inhibiting lung metastasis.^88^ FR17 administration interrupts the activation of fibroblasts and inhibits vascular leakage and angiogenesis induced by tumor‐derived factors in the PMN.^89^ The fusion compound of cytokine LIGHT and vascular‐targeting peptide (LIGHT‐VTP) can normalize tumor blood vessels; furthermore, LIGHT–VTP efficiently targets pathological blood vessels in the PMN, reducing vascular permeability and ECM deposition, thus blocking metastatic lung colonization.^90^ Unfortunately, most of these therapies are in the basic research stage and have not yet entered the preclinical or clinical trials.

### 5.2 Early diagnosis and prediction of cancer

The biological interaction between TME and PMN is increasingly important in tumor progression. Providing an animal model for the PMN is crucial for research and prediction.^91^, ^92^ Using liquid biopsy technology to detect the factors in PMN, exosomes, and CTCs as markers for early diagnosis and prediction of cancer is of great significance. Some studies have confirmed that the expression of Tenascin‐C in regional lymph nodes may be a good predictor of bladder cancer metastasis, providing an important target for early metastasis intervention and treatment.^93^ S100 protein is one of the most important chemokines in the PMN. Therefore, Eisenblaetter et al.^94^ adopted S100A8/A9‐specific single‐photon emission computed tomography probe for the first time to predict the formation of the lung PMN in a syngeneic mouse breast cancer model.

## 6 PERSPECTIVE

Although there are significant advances in the PMN research, other mechanisms of the PMN need to be further studied in the future. Research shows that different tumors can promote the formation of the PMN in metastatic organs by targeting different stromal cells. Better recognition of the role of PMN cells and molecules in different metastatic organs will help prevent early cancer metastasis. The potential research in this field in the future may focus on the following aspects: (1) Do all primary tumors produce the PMN? (2) When the primary tumor forms, does it start to form the PMN? (3) How does the PMN produced by primary tumors change dynamically, and what are the key factors? (4) There is no imaging method that can detect PMN, and it is difficult to obtain premetastatic tissue.

## AUTHOR CONTRIBUTIONS

Hongfei Liu and Guoxin Zhang collected documents and prepared manuscripts. Ran Gao conceptualized, supervised and revised the manuscript. All authors read and approved the final manuscript.

## FUNDING INFORMATION

Chinese Academy of Medical Sciences Innovation Fund for Medical Sciences (2021‐I2M‐1‐013), the National Key Research and Development Program of China (2021YFF0702801, 2022YFF0710705) and the Special Research Fund for Central Universities, Peking Union Medical College (No. 3332022182). Seed Fund for Youth Talent Training Program of Beijing Tongren Hospital Affiliated to Capital Medical University (2020‐YJJ‐ZZL‐034).

## CONFLICT OF INTEREST STATEMENT

The authors declare no competing interests. Ran Gao is an editorial board member of *AMEM* and a coauthor of this article. To minimize bias, she was excluded from all editorial decision making related to the acceptance of this article for publication.

## ETHICS STATEMENT

None.

## References

[1] Chin AR , Wang SE . Cancer tills the Premetastatic field: mechanistic basis and clinical implications. Clin Cancer Res. 2016;22(15):3725‐3733. doi:10.1158/1078-0432.CCR-16-0028 27252414 PMC4970897

[2] van Deventer HW , Palmieri DA , Wu QP , McCook EC , Serody JS . Circulating fibrocytes prepare the lung for cancer metastasis by recruiting Ly‐6C+ monocytes via CCL2. J Immunol. 2013;190(9):4861‐4867. doi:10.4049/jimmunol.1202857 23536638 PMC3740355

[3] Zheng Z , Li YN , Jia S , et al. Lung mesenchymal stromal cells influenced by Th2 cytokines mobilize neutrophils and facilitate metastasis by producing complement C3. Nat Commun. 2021;12(1):6202. doi:10.1038/s41467-021-26460-z 34707103 PMC8551331

[4] Wang D , Sun H , Wei J , Cen B , DuBois RN . CXCL1 is critical for Premetastatic niche formation and metastasis in colorectal cancer. Cancer Res. 2017;77(13):3655‐3665. doi:10.1158/0008-5472.CAN-16-3199 28455419 PMC5877403

[5] Wortzel I , Dror S , Kenific CM , Lyden D . Exosome‐mediated metastasis: communication from a distance. Dev Cell. 2019;49(3):347‐360. doi:10.1016/j.devcel.2019.04.011 31063754

[6] Mathivanan S , Ji H , Simpson RJ . Exosomes: extracellular organelles important in intercellular communication. J Proteomics. 2010;73(10):1907‐1920. doi:10.1016/j.jprot.2010.06.006 20601276

[7] Yoshida K , Tsuda M , Matsumoto R , et al. Exosomes containing ErbB2/CRK induce vascular growth in premetastatic niches and promote metastasis of bladder cancer. Cancer Sci. 2019;110(7):2119‐2132. doi:10.1111/cas.14080 31141251 PMC6609816

[8] Keklikoglou I , Cianciaruso C , Guc E , et al. Chemotherapy elicits pro‐metastatic extracellular vesicles in breast cancer models. Nat Cell Biol. 2019;21(2):190‐202. doi:10.1038/s41556-018-0256-3 30598531 PMC6525097

[9] Zhao S , Mi Y , Zheng B , et al. Highly‐metastatic colorectal cancer cell released miR‐181a‐5p‐rich extracellular vesicles promote liver metastasis by activating hepatic stellate cells and remodelling the tumour microenvironment. J Extracell Vesicles. 2022;11(1):e12186. doi:10.1002/jev2.12186 35041299 PMC8765330

[10] Yuan X , Qian N , Ling S , et al. Breast cancer exosomes contribute to pre‐metastatic niche formation and promote bone metastasis of tumor cells. Theranostics. 2021;11(3):1429‐1445. doi:10.7150/thno.45351 33391543 PMC7738874

[11] Oudin MJ , Jonas O , Kosciuk T , et al. Tumor cell‐driven extracellular matrix remodeling drives Haptotaxis during metastatic progression. Cancer Discov. 2016;6(5):516‐531. doi:10.1158/2159-8290.CD-15-1183 26811325 PMC4854754

[12] Sullivan WJ , Mullen PJ , Schmid EW , et al. Extracellular matrix remodeling regulates glucose metabolism through TXNIP destabilization. Cell. 2018;175(1):117‐132 e21. doi:10.1016/j.cell.2018.08.017 30197082 PMC6151140

[13] Ghoshal A , Rodrigues LC , Gowda CP , et al. Extracellular vesicle‐dependent effect of RNA‐binding protein IGF2BP1 on melanoma metastasis. Oncogene. 2019;38(21):4182‐4196. doi:10.1038/s41388-019-0797-3 30936459 PMC7727312

[14] Deep G , Jain A , Kumar A , et al. Exosomes secreted by prostate cancer cells under hypoxia promote matrix metalloproteinases activity at pre‐metastatic niches. Mol Carcinog. 2020;59(3):323‐332. doi:10.1002/mc.23157 31943365 PMC7189745

[15] Wu S , Xing X , Wang Y , et al. The pathological significance of LOXL2 in pre‐metastatic niche formation of HCC and its related molecular mechanism. Eur J Cancer. 2021;147:63‐73. doi:10.1016/j.ejca.2021.01.011 33618200

[16] Ratajczak‐Wielgomas K , Dziegiel P . The role of periostin in neoplastic processes. Folia Histochem Cytobiol. 2015;53(2):120‐132. doi:10.5603/FHC.a2015.0014 26150285

[17] Wang Z , Xiong S , Mao Y , et al. Periostin promotes immunosuppressive premetastatic niche formation to facilitate breast tumour metastasis. J Pathol. 2016;239(4):484‐495. doi:10.1002/path.4747 27193093

[18] Cheung KJ , Ewald AJ . A collective route to metastasis: seeding by tumor cell clusters. Science. 2016;352(6282):167‐169. doi:10.1126/science.aaf6546 27124449 PMC8183671

[19] Lo HC , Xu Z , Kim IS , et al. Resistance to natural killer cell immunosurveillance confers a selective advantage to polyclonal metastasis. Nat Cancer. 2020;1(7):709‐722. doi:10.1038/s43018-020-0068-9 35122036

[20] Peinado H , Zhang H , Matei IR , et al. Pre‐metastatic niches: organ‐specific homes for metastases. Nat Rev Cancer. 2017;17(5):302‐317. doi:10.1038/nrc.2017.6 28303905

[21] Doglioni G , Parik S , Fendt SM . Interactions in the (pre)metastatic niche support metastasis formation. Front Oncol. 2019;9:219. doi:10.3389/fonc.2019.00219 31069166 PMC6491570

[22] Liu Y , Cao X . Characteristics and significance of the pre‐metastatic niche. Cancer Cell. 2016;30(5):668‐681. doi:10.1016/j.ccell.2016.09.011 27846389

[23] Yang WW , Yang LQ , Zhao F , et al. Epiregulin promotes lung metastasis of salivary adenoid cystic carcinoma. Theranostics. 2017;7(15):3700‐3714. doi:10.7150/thno.19712 29109770 PMC5667342

[24] Zeng Z , Li Y , Pan Y , et al. Cancer‐derived exosomal miR‐25‐3p promotes pre‐metastatic niche formation by inducing vascular permeability and angiogenesis. Nat Commun. 2018;9(1):5395. doi:10.1038/s41467-018-07810-w 30568162 PMC6300604

[25] Peinado H , Aleckovic M , Lavotshkin S , et al. Melanoma exosomes educate bone marrow progenitor cells toward a pro‐metastatic phenotype through MET. Nat Med. 2012;18(6):883‐891. doi:10.1038/nm.2753 22635005 PMC3645291

[26] Duan S , Nordmeier S , Byrnes AE , Buxton ILO . Extracellular vesicle‐mediated purinergic signaling contributes to host microenvironment plasticity and metastasis in triple negative breast cancer. Int J Mol Sci. 2021;22(2):597. doi:10.3390/ijms22020597 33435297 PMC7827112

[27] Clark AG , Vignjevic DM . Modes of cancer cell invasion and the role of the microenvironment. Curr Opin Cell Biol. 2015;36:13‐22. doi:10.1016/j.ceb.2015.06.004 26183445

[28] Zhao J , Schlosser HA , Wang Z , et al. Tumor‐derived extracellular vesicles inhibit natural killer cell function in pancreatic cancer. Cancers (Basel). 2019;11(6):874. doi:10.3390/cancers11060874 31234517 PMC6628179

[29] Jing B , Wang T , Sun B , et al. IL6/STAT3 signaling orchestrates Premetastatic niche formation and immunosuppressive traits in lung. Cancer Res. 2020;80(4):784‐797. doi:10.1158/0008-5472.CAN-19-2013 31848193

[30] Leary N , Walser S , He Y , et al. Melanoma‐derived extracellular vesicles mediate lymphatic remodelling and impair tumour immunity in draining lymph nodes. J Extracell Vesicles. 2022;11(2):e12197. doi:10.1002/jev2.12197 35188342 PMC8859913

[31] Wang M , Qin Z , Wan J , et al. Tumor‐derived exosomes drive pre‐metastatic niche formation in lung via modulating CCL1(+) fibroblast and CCR8(+) Treg cell interactions. Cancer Immunol Immunother. 2022;71(11):2717‐2730. doi:10.1007/s00262-022-03196-3 35428909 PMC10992578

[32] Grivennikov SI , Greten FR , Karin M . Immunity, inflammation, and cancer. Cell. 2010;140(6):883‐899. doi:10.1016/j.cell.2010.01.025 20303878 PMC2866629

[33] Rodrigues G , Hoshino A , Kenific CM , et al. Tumour exosomal CEMIP protein promotes cancer cell colonization in brain metastasis. Nat Cell Biol. 2019;21(11):1403‐1412. doi:10.1038/s41556-019-0404-4 31685984 PMC7354005

[34] Nagahashi M , Yamada A , Katsuta E , et al. Targeting the SphK1/S1P/S1PR1 Axis that links obesity, chronic inflammation, and breast cancer metastasis. Cancer Res. 2018;78(7):1713‐1725. doi:10.1158/0008-5472.CAN-17-1423 29351902 PMC6945803

[35] Liu Y , Gu Y , Han Y , et al. Tumor Exosomal RNAs promote lung pre‐metastatic niche formation by activating alveolar epithelial TLR3 to recruit neutrophils. Cancer Cell. 2016;30(2):243‐256. doi:10.1016/j.ccell.2016.06.021 27505671

[36] Wculek SK , Malanchi I . Neutrophils support lung colonization of metastasis‐initiating breast cancer cells. Nature. 2015;528(7582):413‐417. doi:10.1038/nature16140 26649828 PMC4700594

[37] Bald T , Quast T , Landsberg J , et al. Ultraviolet‐radiation‐induced inflammation promotes angiotropism and metastasis in melanoma. Nature. 2014;507(7490):109‐113. doi:10.1038/nature13111 24572365

[38] Hoshino A , Costa‐Silva B , Shen TL , et al. Tumour exosome integrins determine organotropic metastasis. Nature. 2015;527(7578):329‐335. doi:10.1038/nature15756 26524530 PMC4788391

[39] Francia G , Cruz‐Munoz W , Man S , Xu P , Kerbel RS . Mouse models of advanced spontaneous metastasis for experimental therapeutics. Nat Rev Cancer. 2011;11(2):135‐141. doi:10.1038/nrc3001 21258397 PMC4540342

[40] Yang C , Wang Z , Li L , et al. Aged neutrophils form mitochondria‐dependent vital NETs to promote breast cancer lung metastasis. J Immunother Cancer. 2021;9(10):e002875. doi:10.1136/jitc-2021-002875 34716206 PMC8559246

[41] Tyagi A , Wu SY , Sharma S , et al. Exosomal miR‐4466 from nicotine‐activated neutrophils promotes tumor cell stemness and metabolism in lung cancer metastasis. Oncogene. 2022;41(22):3079‐3092. doi:10.1038/s41388-022-02322-w 35461327 PMC9135627

[42] Mundy GR . Metastasis to bone: causes, consequences and therapeutic opportunities. Nat Rev Cancer. 2002;2(8):584‐593. doi:10.1038/nrc867 12154351

[43] Cox TR , Rumney RMH , Schoof EM , et al. The hypoxic cancer secretome induces pre‐metastatic bone lesions through lysyl oxidase. Nature. 2015;522(7554):106‐110. doi:10.1038/nature14492 26017313 PMC4961239

[44] Zhao E , Wang L , Dai J , et al. Regulatory T cells in the bone marrow microenvironment in patients with prostate cancer. Oncoimmunology. 2012;1(2):152‐161. doi:10.4161/onci.1.2.18480 22720236 PMC3376984

[45] Chu GC , Zhau HE , Wang R , et al. RANK‐ and c‐met‐mediated signal network promotes prostate cancer metastatic colonization. Endocr Relat Cancer. 2014;21(2):311‐326. doi:10.1530/ERC-13-0548 24478054 PMC3959765

[46] Wu K , Feng J , Lyu F , et al. Exosomal miR‐19a and IBSP cooperate to induce osteolytic bone metastasis of estrogen receptor‐positive breast cancer. Nat Commun. 2021;12(1):5196. doi:10.1038/s41467-021-25473-y 34465793 PMC8408156

[47] Guo L , Zhu Y , Li L , et al. Breast cancer cell‐derived exosomal miR‐20a‐5p promotes the proliferation and differentiation of osteoclasts by targeting SRCIN1. Cancer Med. 2019;8(12):5687‐5701. doi:10.1002/cam4.2454 31385464 PMC6745844

[48] Costa‐Silva B , Aiello NM , Ocean AJ , et al. Pancreatic cancer exosomes initiate pre‐metastatic niche formation in the liver. Nat Cell Biol. 2015;17(6):816‐826. doi:10.1038/ncb3169 25985394 PMC5769922

[49] Wang Q , Ni Q , Wang X , Zhu H , Wang Z , Huang J . High expression of RAB27A and TP53 in pancreatic cancer predicts poor survival. Med Oncol. 2015;32(1):372. doi:10.1007/s12032-014-0372-2 25428385

[50] Kren N , Michaud D , Bagchi S , Greene K , Pylayeva‐Gupta Y . Rab27a plays a dual role in metastatic propensity of pancreatic cancer. Sci Rep. 2020;10(1):7390. doi:10.1038/s41598-020-64248-1 32355248 PMC7193593

[51] Fong MY , Zhou W , Liu L , et al. Breast‐cancer‐secreted miR‐122 reprograms glucose metabolism in premetastatic niche to promote metastasis. Nat Cell Biol. 2015;17(2):183‐194. doi:10.1038/ncb3094 25621950 PMC4380143

[52] Morrissey SM , Zhang F , Ding C , et al. Tumor‐derived exosomes drive immunosuppressive macrophages in a pre‐metastatic niche through glycolytic dominant metabolic reprogramming. Cell Metab. 2021;33(10):2040‐2058 e10. doi:10.1016/j.cmet.2021.09.002 34559989 PMC8506837

[53] Zhang C , Wang XY , Zhang P , et al. Cancer‐derived exosomal HSPC111 promotes colorectal cancer liver metastasis by reprogramming lipid metabolism in cancer‐associated fibroblasts. Cell Death Dis. 2022;13(1):57. doi:10.1038/s41419-022-04506-4 35027547 PMC8758774

[54] Zeng Z , Xu S , Wang F , et al. HAO1‐mediated oxalate metabolism promotes lung pre‐metastatic niche formation by inducing neutrophil extracellular traps. Oncogene. 2022;41(29):3719‐3731. doi:10.1038/s41388-022-02248-3 35739335 PMC9287177

[55] Ordonez‐Moran P , Huelsken J . Complex metastatic niches: already a target for therapy? Curr Opin Cell Biol. 2014;31:29‐38. doi:10.1016/j.ceb.2014.06.012 25036901

[56] Bonapace L , Coissieux MM , Wyckoff J , et al. Cessation of CCL2 inhibition accelerates breast cancer metastasis by promoting angiogenesis. Nature. 2014;515(7525):130‐133. doi:10.1038/nature13862 25337873

[57] Gabrilovich DI , Ostrand‐Rosenberg S , Bronte V . Coordinated regulation of myeloid cells by tumours. Nat Rev Immunol. 2012;12(4):253‐268. doi:10.1038/nri3175 22437938 PMC3587148

[58] Deguchi A , Tomita T , Ohto U , et al. Eritoran inhibits S100A8‐mediated TLR4/MD‐2 activation and tumor growth by changing the immune microenvironment. Oncogene. 2016;35(11):1445‐1456. doi:10.1038/onc.2015.211 26165843

[59] Catena R , Bhattacharya N , El Rayes T , et al. Bone marrow‐derived Gr1+ cells can generate a metastasis‐resistant microenvironment via induced secretion of thrombospondin‐1. Cancer Discov. 2013;3(5):578‐589. doi:10.1158/2159-8290.CD-12-0476 23633432 PMC3672408

[60] Yan HH , Pickup M , Pang Y , et al. Gr‐1+CD11b+ myeloid cells tip the balance of immune protection to tumor promotion in the premetastatic lung. Cancer Res. 2010;70(15):6139‐6149. doi:10.1158/0008-5472.CAN-10-0706 20631080 PMC4675145

[61] Long Y , Lu Z , Xu S , et al. Self‐delivery micellar nanoparticles prevent Premetastatic niche formation by interfering with the early recruitment and vascular destruction of granulocytic myeloid‐derived suppressor cells. Nano Lett. 2020;20(4):2219‐2229. doi:10.1021/acs.nanolett.9b03883 31823615

[62] Sceneay J , Chow MT , Chen A , et al. Primary tumor hypoxia recruits CD11b+/Ly6Cmed/Ly6G+ immune suppressor cells and compromises NK cell cytotoxicity in the premetastatic niche. Cancer Res. 2012;72(16):3906‐3911. doi:10.1158/0008-5472.CAN-11-3873 22751463

[63] Zhou X , Yan T , Huang C , et al. Melanoma cell‐secreted exosomal miR‐155‐5p induce proangiogenic switch of cancer‐associated fibroblasts via SOCS1/JAK2/STAT3 signaling pathway. J Exp Clin Cancer Res. 2018;37(1):242. doi:10.1186/s13046-018-0911-3 30285793 PMC6169013

[64] Pein M , Insua‐Rodriguez J , Hongu T , et al. Metastasis‐initiating cells induce and exploit a fibroblast niche to fuel malignant colonization of the lungs. Nat Commun. 2020;11(1):1494. doi:10.1038/s41467-020-15188-x 32198421 PMC7083860

[65] Zeng H , Hou Y , Zhou X , et al. Cancer‐associated fibroblasts facilitate premetastatic niche formation through lncRNA SNHG5‐mediated angiogenesis and vascular permeability in breast cancer. Theranostics. 2022;12(17):7351‐7370. doi:10.7150/thno.74753 36438499 PMC9691361

[66] Mao X , Tey SK , Yeung CLS , et al. Nidogen 1‐enriched extracellular vesicles facilitate extrahepatic metastasis of liver cancer by activating pulmonary fibroblasts to secrete tumor necrosis factor receptor 1. Adv Sci (Weinh). 2020;7(21):2002157. doi:10.1002/advs.202002157 33173740 PMC7640351

[67] Ji Q , Zhou L , Sui H , et al. Primary tumors release ITGBL1‐rich extracellular vesicles to promote distal metastatic tumor growth through fibroblast‐niche formation. Nat Commun. 2020;11(1):1211. doi:10.1038/s41467-020-14869-x 32139701 PMC7058049

[68] Gong Z , Li Q , Shi J , et al. Lung fibroblasts facilitate pre‐metastatic niche formation by remodeling the local immune microenvironment. Immunity. 2022;55(8):1483‐1500 e9. doi:10.1016/j.immuni.2022.07.001 35908547 PMC9830653

[69] Binnewies M , Pollack JL , Rudolph J , et al. Targeting TREM2 on tumor‐associated macrophages enhances immunotherapy. Cell Rep. 2021;37(3):109844. doi:10.1016/j.celrep.2021.109844 34686340

[70] Zhao S , Mi Y , Guan B , et al. Tumor‐derived exosomal miR‐934 induces macrophage M2 polarization to promote liver metastasis of colorectal cancer. J Hematol Oncol. 2020;13(1):156. doi:10.1186/s13045-020-00991-2 33213490 PMC7678301

[71] Qiu S , Xie L , Lu C , et al. Gastric cancer‐derived exosomal miR‐519a‐3p promotes liver metastasis by inducing intrahepatic M2‐like macrophage‐mediated angiogenesis. J Exp Clin Cancer Res. 2022;41(1):296. doi:10.1186/s13046-022-02499-8 36217165 PMC9549645

[72] Gentles AJ , Newman AM , Liu CL , et al. The prognostic landscape of genes and infiltrating immune cells across human cancers. Nat Med. 2015;21(8):938‐945. doi:10.1038/nm.3909 26193342 PMC4852857

[73] Moresco MA , Raccosta L , Corna G , et al. Enzymatic inactivation of oxysterols in breast tumor cells constraints metastasis formation by reprogramming the metastatic lung microenvironment. Front Immunol. 2018;9:2251. doi:10.3389/fimmu.2018.02251 30333826 PMC6176086

[74] Wu CF , Andzinski L , Kasnitz N , et al. The lack of type I interferon induces neutrophil‐mediated pre‐metastatic niche formation in the mouse lung. Int J Cancer. 2015;137(4):837‐847. doi:10.1002/ijc.29444 25604426

[75] Lee W , Ko SY , Mohamed MS , Kenny HA , Lengyel E , Naora H . Neutrophils facilitate ovarian cancer premetastatic niche formation in the omentum. J Exp Med. 2019;216(1):176‐194. doi:10.1084/jem.20181170 30567719 PMC6314534

[76] De Meo ML , Spicer JD . The role of neutrophil extracellular traps in cancer progression and metastasis. Semin Immunol. 2021;57:101595. doi:10.1016/j.smim.2022.101595 35125298

[77] Jackstadt R , van Hooff SR , Leach JD , et al. Epithelial NOTCH signaling rewires the tumor microenvironment of colorectal cancer to drive poor‐prognosis subtypes and metastasis. Cancer Cell. 2019;36(3):319‐336 e7. doi:10.1016/j.ccell.2019.08.003 31526760 PMC6853173

[78] Qi M , Xia Y , Wu Y , et al. Lin28B‐high breast cancer cells promote immune suppression in the lung pre‐metastatic niche via exosomes and support cancer progression. Nat Commun. 2022;13(1):897. doi:10.1038/s41467-022-28438-x 35173168 PMC8850492

[79] Coffelt SB , Kersten K , Doornebal CW , et al. IL‐17‐producing gammadelta T cells and neutrophils conspire to promote breast cancer metastasis. Nature. 2015;522(7556):345‐348. doi:10.1038/nature14282 25822788 PMC4475637

[80] Reymond N , d'Agua BB , Ridley AJ . Crossing the endothelial barrier during metastasis. Nat Rev Cancer. 2013;13(12):858‐870. doi:10.1038/nrc3628 24263189

[81] Franses JW , Drosu NC , Gibson WJ , Chitalia VC , Edelman ER . Dysfunctional endothelial cells directly stimulate cancer inflammation and metastasis. Int J Cancer. 2013;133(6):1334‐1344. doi:10.1002/ijc.28146 23463345 PMC3707950

[82] Bertocchi A , Carloni S , Ravenda PS , et al. Gut vascular barrier impairment leads to intestinal bacteria dissemination and colorectal cancer metastasis to liver. Cancer Cell. 2021;39(5):708‐724 e11. doi:10.1016/j.ccell.2021.03.004 33798472

[83] Zhang G , Li M , Zhou D , Yang X , Zhang W , Gao R . Loss of endothelial EMCN drives tumor lung metastasis through the premetastatic niche. J Transl Med. 2022;20(1):446. doi:10.1186/s12967-022-03649-4 36184589 PMC9528146

[84] Bertolini G , Compagno M , Belisario DC , et al. CD73/adenosine pathway involvement in the interaction of non‐small cell lung cancer stem cells and bone cells in the pre‐metastatic niche. Int J Mol Sci. 2022;23(9):5126. doi:10.3390/ijms23095126 35563517 PMC9104817

[85] Narasimhan H , Ferraro F , Bleilevens A , Weiskirchen R , Stickeler E , Maurer J . Tumor necrosis factor‐alpha (TNFalpha) stimulate triple‐negative breast cancer stem cells to promote Intratumoral invasion and Neovasculogenesis in the liver of a xenograft model. Biology (Basel). 2022;11(10):1481. doi:10.3390/biology11101481 36290384 PMC9598572

[86] Lu Z , Liu H , Ma L , et al. Micellar nanoparticles inhibit breast cancer and pulmonary metastasis by modulating the recruitment and depletion of myeloid‐derived suppressor cells. Nanoscale. 2022;14(46):17315‐17330. doi:10.1039/d2nr03880c 36374496

[87] Kranz LM , Diken M , Haas H , et al. Systemic RNA delivery to dendritic cells exploits antiviral defence for cancer immunotherapy. Nature. 2016;534(7607):396‐401. doi:10.1038/nature18300 27281205

[88] Yan Y , Du C , Duan X , et al. Inhibiting collagen I production and tumor cell colonization in the lung via miR‐29a‐3p loading of exosome−/liposome‐based nanovesicles. Acta Pharm Sin B. 2022;12(2):939‐951. doi:10.1016/j.apsb.2021.08.011 35256956 PMC8897025

[89] Zhou Y , Ke P , Bao X , et al. Peptide nano‐blanket impedes fibroblasts activation and subsequent formation of pre‐metastatic niche. Nat Commun. 2022;13(1):2906. doi:10.1038/s41467-022-30634-8 35614076 PMC9132894

[90] He B , Johansson‐Percival A , Backhouse J , et al. Remodeling of metastatic vasculature reduces lung colonization and sensitizes overt metastases to immunotherapy. Cell Rep. 2020;30(3):714‐724 e5. doi:10.1016/j.celrep.2019.12.013 31968248

[91] Sajjad H , Imtiaz S , Noor T , Siddiqui YH , Sajjad A , Zia M . Cancer models in preclinical research: a chronicle review of advancement in effective cancer research. Animal Model Exp Med. 2021;4(2):87‐103. doi:10.1002/ame2.12165 34179717 PMC8212826

[92] Patten LW , Blatchford P , Strand M , Kaizer AM . Assessing the performance of different outcomes for tumor growth studies with animal models. Animal Model Exp Med. 2022;5(3):248‐257. doi:10.1002/ame2.12250 35699330 PMC9240739

[93] Silvers CR , Messing EM , Miyamoto H , Lee YF . Tenascin‐C expression in the lymph node pre‐metastatic niche in muscle‐invasive bladder cancer. Br J Cancer. 2021;125(10):1399‐1407. doi:10.1038/s41416-021-01554-z 34564696 PMC8575937

[94] Sakaguchi M . S100‐SPECT uncovers cellular and molecular events of pre‐metastatic niche formation and following organ‐specific cancer metastasis. Theranostics. 2017;7(10):2649‐2651. doi:10.7150/thno.19866 28819453 PMC5558559

